# ﻿*Ajaniaflavida* (Asteraceae, Anthemideae), a distinct new species from southwestern China

**DOI:** 10.3897/phytokeys.239.119028

**Published:** 2024-03-19

**Authors:** Xiao-Rui Chi, Hai-Song Wu, Long Wang

**Affiliations:** 1 Key Laboratory of Plant Resources Conservation and Sustainable Utilization, South China Botanical Garden, Chinese Academy of Sciences, Guangzhou 510650, Guangdong, China South China Botanical Garden, Chinese Academy of Sciences Guangzhou China; 2 University of Chinese Academy of Sciences, Beijing 100049, China University of Chinese Academy of Sciences Beijing China

**Keywords:** Compositae, taxonomy, Yangtze River

## Abstract

*Ajaniaflavida*, a new species from western Sichuan and eastern Xizang, China, is described and illustrated. It is readily assigned to A.sect.Ajania owing to its straw-colored, glossy involucres and marginally whitish scarious phyllaries. Within the section, it is distinct in being a shrub of 1−2 m in height, and in having creamy yellow florets. It is superficially similar to *A.ramosa* in A.sect.Phaeoscyphus, but can easily be distinguished by, among other characters, the plant height, color of the florets and margins of the phyllaries. In addition, we provide a distribution map of the new species.

## ﻿Introduction

*Ajania* Poljak. (Asteraceae, Anthemideae) consists of 30−39 species mainly distributed in central Asia (Bremer and Humphries 1993; Oberprieler et al. 2007a, b, 2009; Shih et al. 2011). In China, 35 species are currently recognized, among which 23 are endemic (Shih et al. 2011). Over the past decade, taxonomic updates in this genus have been infrequent, with only a new species described from Iran (Sonboli et al. 2013).

**Figure 1.**
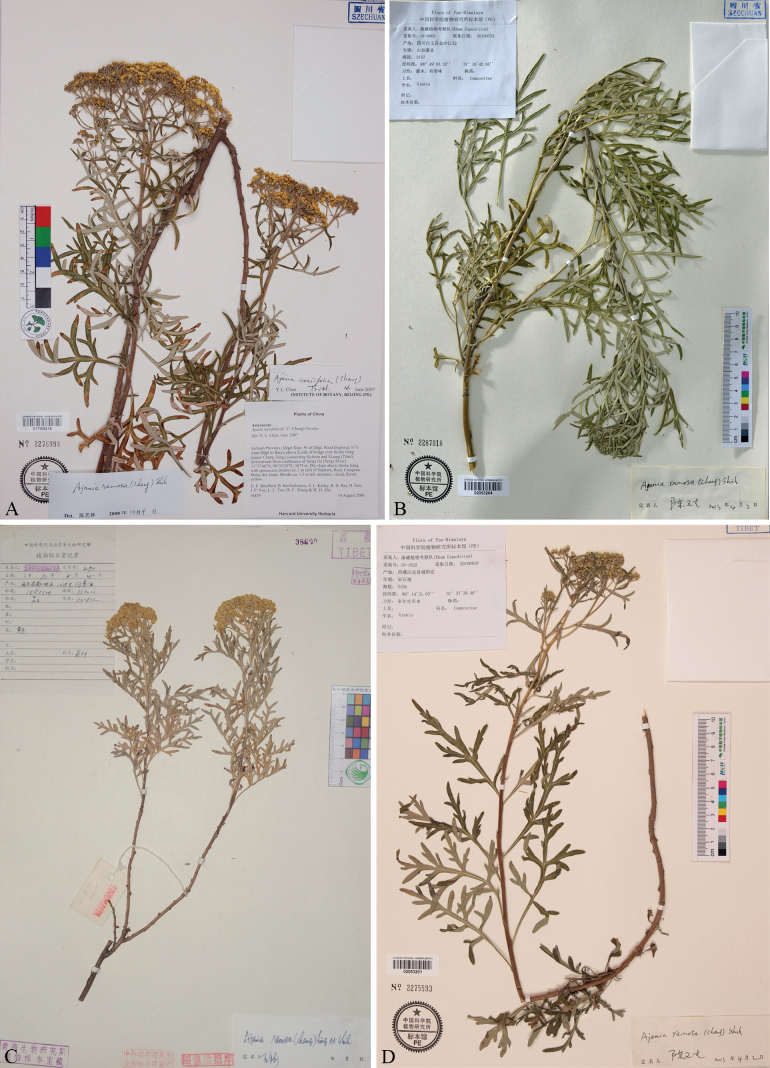
Specimens of *Ajaniaflavida* sp. nov. previously misidentified as *A.ramosa* or *A.variifolia***A** China, Sichuan, Dêgê, *D. E. Boufford et al. 36429* (PE) **B** China, Sichuan, Baiyü, *Kham Exped. 10-0662* (PE) **C** China, Xizang, Jomda, *HNWP Xizang Exped. 2195* (HNWP) **D** China, Xizang, Jomda, *Kham Exped. 10-1622* (PE).

**Figure 2.**
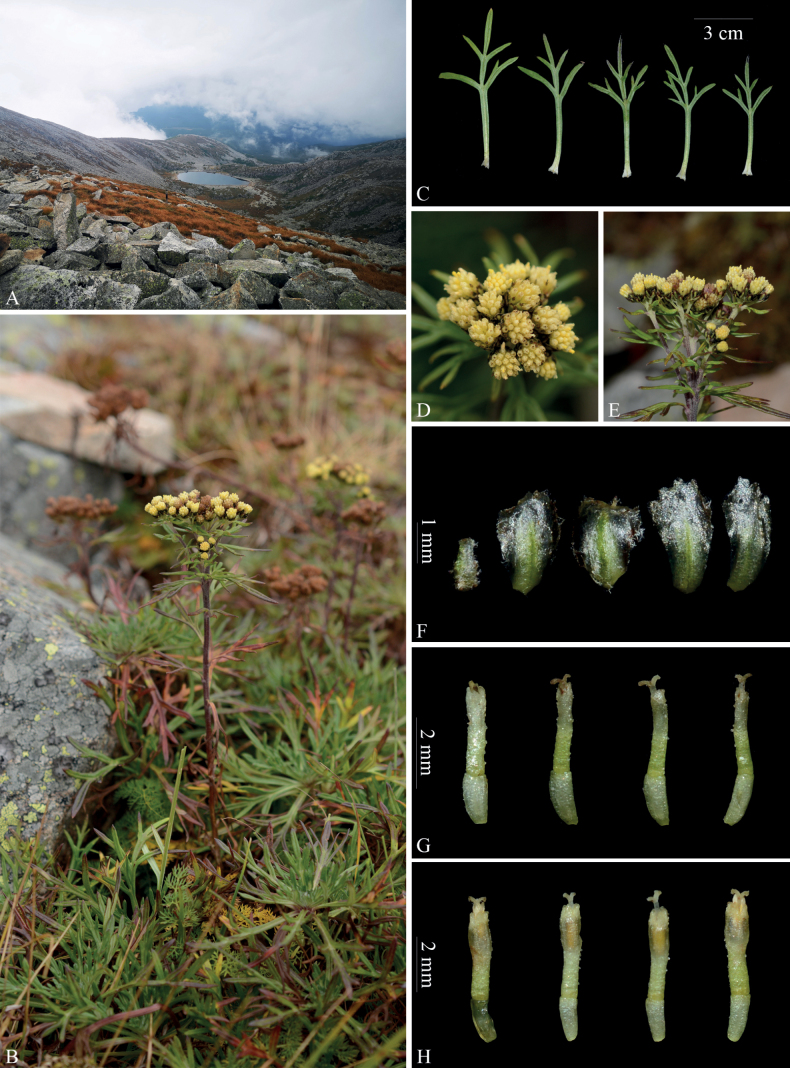
*Ajaniaramosa* in the wild (China, Shaanxi, Meixian) **A** habitat **B** habit **C** basal leaves (adaxial surface) **D** synflorescence (top view) **E** synflorescence (side view) **F** phyllaries (abaxial surface) **G** marginal female florets **H** central disk florets. Photographs by Long Wang.

During herbarium surveys of *Ajania* in China, seven collections, including *D. E. Boufford et al. 36429* (F, PE; Fig. 1A), *Kham Exped. 10-0662* (PE; Fig. 1B), *Y. W. Tsui 5942* (PE), and *M. Z. Wen & S. C. Xiao Xiang157* (CDBI) from Sichuan, and *HNWP Xizang Exped. 2195* (HNWP; Fig. 1C), *Kham Exped. 10-1622* (PE; Fig. 1D) and *Qinghai-Xizang Vegetat. Exped. 9655* (PE) from Xizang, all in China, caught our attention. Most of these collections have been previously identified as *A.ramosa* (C. C. Chang) C. Shih (Fig. 2), but they are quite different from that species in an array of characters. Plants of this taxon are shrubs of 1−2 m tall, with leaf blades 2-pinnatisect, involucres ca. 3 mm in diameter, and margins of the phyllaries whitish scarious, while in *A.ramosa* the plants are subshrubs of 40−60 cm tall, with leaf blades 1(−2)-pinnatisect, involucres 4−5 mm in diameter, and margins of the phyllaries brown scarious. To precisely determine the identity of these collections, we undertook a field visit to Jomda in eastern Xizang in September 2019. Through careful comparisons, we found that this taxon is indeed quite different from *A.ramosa* in A.sect.Phaeoscyphus, which is characterized by having larger (4−10 mm in diameter) capitula, not straw-colored, not glossy involucres and marginally dark brown or purple scarious phyllaries. It can be, however, readily referred to A.sectAjania based on the smaller (ca. 3 mm in diameter) capitula, straw-colored, glossy involucres and marginally whitish scarious phyllaries. It is easily distinguishable from the remaining species within this section in being shrubs of 1−2 m tall and having creamy yellow florets. The taxon in question therefore represents a hitherto undescribed species, which we describe below.

## ﻿Material and methods

For morphological comparisons, we examined physical or digitalized herbarium specimens (with high-resolution) of *Ajania* deposited at several major herbaria in China, including CDBI, HNWP, IBSC, KUN, NAS, PE, SZ, and WUK (acronyms follow Thiers (2024)). Specimens of *A.flavida* were collected and photographed during our field investigation to Xizang Autonomous Region in 2019. Morphological observations and measurements were based on fresh material as well as all herbarium specimens of this species. For conservation assessment, we used Geo CAT, the online geospatial conservation assessment tool (Bachman et al. 2011; http://geocat.kew.org/) to calculate the Extent of Occurrence (EOO) and Area of Occupancy (AOO) with a user-defined cell width of 2 km.

## ﻿Taxonomic treatment

### ﻿ Ajania flavida

Taxon classificationPlantaeAsteralesAsteraceae

Long Wang sp. nov.

74A7A89E-3B7E-5C27-B3C0-2746FE3FE91E

urn:lsid:ipni.org:names:77338638-1

Figs 1 , 3 , 4

#### Diagnosis.

*Ajaniaflavida* is distinct in A.sect.Ajania in being shrubs of 1−2 m in height and having creamy yellow florets. It is superficially similar to *A.ramosa* in A.sect.Phaeoscyphus, but can be easily distinguished by the plant habit (shrub vs. subshrub), plant height (1−2 m vs. 40−60 cm), leaf division (2-pinnatisect vs. 1(−2)-pinnatisect), size of the involucres (ca. 3 mm vs. 4−5 mm in diameter), color (creamy yellow vs. yellow) of the florets and margins (whitish scarious vs. brown scarious) of the phyllaries.

**Figure 3.**
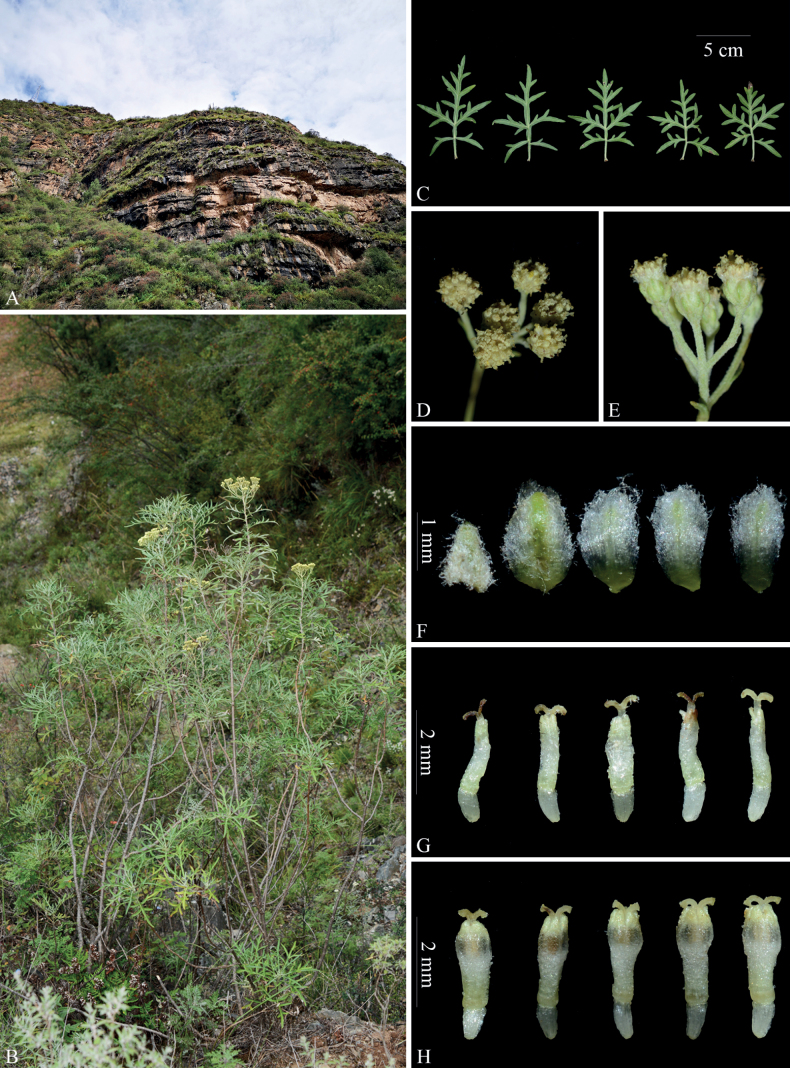
*Ajaniaflavida* sp. nov. in the wild (China, Xizang, Jomda) **A** habitat **B** habit **C** basal leaves (adaxial surface) **D** synflorescence (top view) **E** synflorescence (side view) **F** phyllaries (abaxial surface) **G** marginal female florets **H** central disk florets. Photographs by Long Wang.

#### Type.

**Figure 4.**
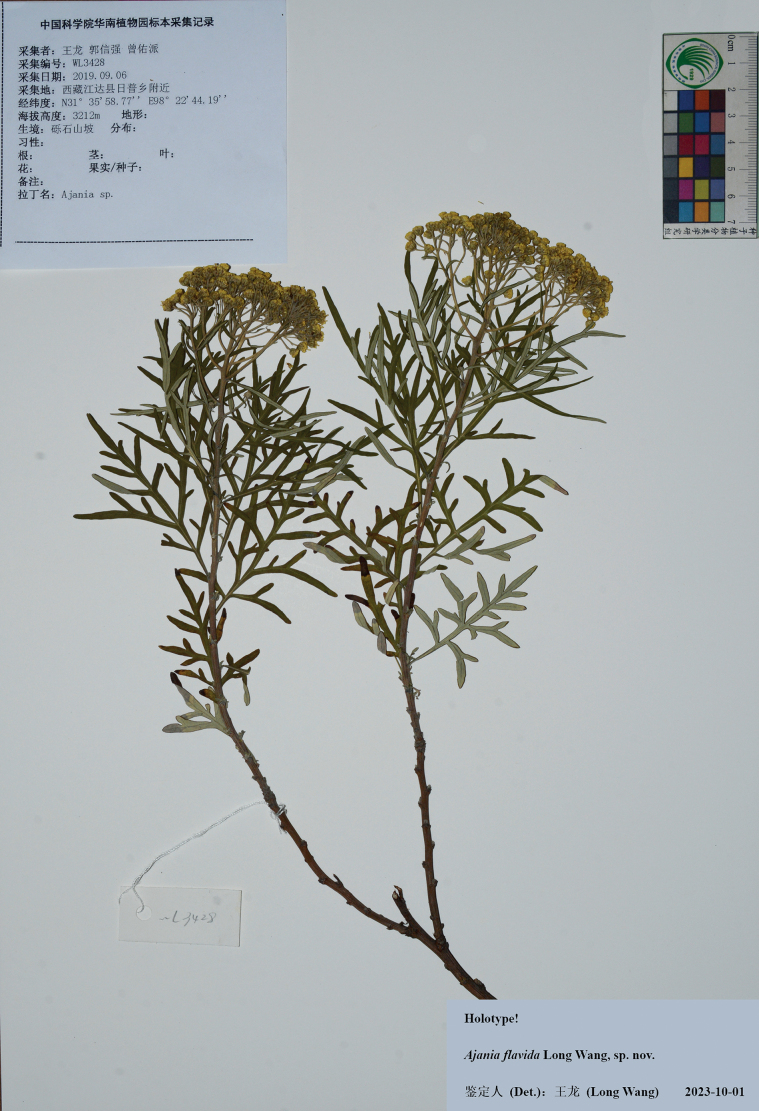
Holotype sheet of *Ajaniaflavida* sp. nov.

China. Xizang: Jomda, Tongpu, 31°35'58.77"N, 98°22'44.19"E, rocky slopes along river, 3212 m a.s.l., 6 September 2019 (fl.), *Long Wang, Xin-qiang Guo & You-pai Zeng 3428* (holotype: IBSC; isotypes: IBSC). Fig. 4.

#### Description.

Shrubs, 1–2 m tall. Old branches gray-black, sparsely sericeous or glabrescent, with densely leafy tips; flowering branches robust, densely grey powdery-sericeous, especially in upper parts and on peduncles. Proximal leaves of flowering branches withered during anthesis. Middle leaves of flowering branches shortly petiolate; petiole 1–2 cm long; leaf blade ovate in outline, 7–10 cm long, 6–7 cm wide, adaxially dark green, grey sericeous, abaxially greyish, densely grey sericeous, 2-pinnatisect; lobes usually 5, narrowly oblong, lateral 1 or 2 pairs 3–4 cm long, 1.0–2.5 cm wide, distal ones 4–6 cm long, 2–3 cm wide, 1-pinnatisect; segments 2–7, lanceolate to narrowly oblong, lateral 1–3 pairs 4–20 mm long, 2–4 mm wide, distal ones 2–4 cm long, 2–5 mm wide. Distal leaves of flowering branches shortly petiolate to sessile; petiole, when present, 0.5–1.5 cm long; leaf blade ovate to linear in outline, progressively smaller upward, 1-pinnatisect to undivided. Synflorescence a terminal compound flat-topped panicle, 5–10 cm in diameter. Capitula many, erect. Involucres campanulate, ca. 3 mm in diameter, outside straw-colored, glossy; phyllaries in 4 rows, outer ones ovate to triangular-ovate, 1.0–1.2 mm long, 0.8–1.0 mm wide, abaxially densely whitish sericeous, apex acute, middle ones oblong to elliptic, 1.5–2.0 mm long, 1.0–1.2 mm wide, abaxially whitish sericeous, margin narrowly to broadly whitish scarious, apex rounded, inner ones narrowly oblong to oblong, 1.5–2 mm long, 0.6–1 mm wide, abaxially slightly whitish sericeous, margin broadly whitish scarious, apex rounded to obtuse. Florets creamy yellow, exterior with several sessile glands. Marginal female florets 9–11, 2–2.8 mm long; tube 0.4–0.7 mm long; corolla narrowly tubular, 0.8–1.1 mm long, apically 4–5-denticulate. Central disk florets many, 2.8–3.2 mm long; tube 0.8–1.2 mm long; corolla broadly tubular, 0.9–1.2 mm long, apically 5-denticulate, incurved; style 1.1–1.3 mm long; branches creamy yellow. Achenes (immature) 0.6–0.8 mm long, obconic. Pappus absent.

#### Distribution and habitat.

**Figure 5.**
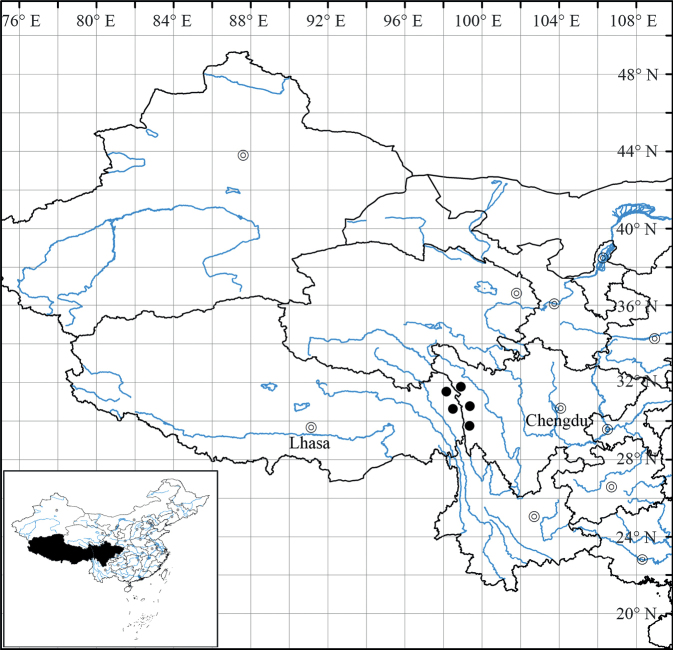
Distribution of *Ajaniaflavida* sp. nov. (Black dot).

*Ajaniaflavida* is currently known only from western Sichuan (Baiyü, Batang, Dêgê) and eastern Xizang (Gonjo, Jomda), China (Fig. 5). It grows on rocky slopes in gorges at elevations of 3075–3800 m above sea level.

#### Etymology.

The specific epithet ‘*flavida*’ refers to the creamy yellow florets of this new species.

#### Phenology.

Flowering in September; fruiting in October.

#### Vernacular name.

川藏亚菊 (Chinese pinyin: chuān zàng yà jú).

#### Conservation status.

*Ajaniaflavida* is currently known only from eight collections made from western Sichuan (Baiyü, Batang, Dêgê) and eastern Xizang (Gonjo, Jomda), China. It usually grows on rocky slopes along roadsides in the gorges, and its habitat is at risk through human activities, such as overgrazing and road construction (Wang, pers. obs.). The Extent of Occurrence (EOO) and the Area of Occupancy (AOO) are calculated to be 7516.23 km^2^ and 32 km^2^, respectively. According to the IUCN Red List Categories and Criteria (IUCN 2012, 2022), this species should be categorized as Vulnerable (VU): B1ab(iii)+2ab(iii).

#### Notes.

Two collections of *Ajaniaflavida*, viz. *D. E. Boufford et al. 36429* (F, PE) and *M. Z. Wen & S. C. Xiao Xiang157* (CDBI), have been previously misidentified as *A.variifolia* C. C. Chang, a species occurring in Heilongjiang, Hubei and Shaanxi in China. *Ajaniaflavida* is readily distinguished from *A.variifolia* in being a shrub (vs. subshrub), 1−2 m (30−60 cm) tall and having leaf blades 2-pinnatisect (vs. 1-pinnatisect), involucres ca. 3 mm (vs. 4−5 mm) in diameter, and margins of phyllaries whitish (vs. brown) scarious.

It is noteworthy that the identity of *Ajaniaramosa* needs to be further determined. According to our observations on both herbarium specimens and living plants in the wild, this species is poorly defined. It may encompass elements of multiple species in the genus.

#### Additional specimens examined

**(paratypes).** China. Sichuan: Baiyü, Jinsha xiang, by the Yangtze River, among scrub in valley, 3157 m, 31°16'42.66"N, 98°49'03.32"E, 25 July 2010, *Kham Exped. 10-0662* (PE); Batang, Lieyi xiang, Yidun, on rocks along road, 3400 m, 19 September 1984, *M. Z. Wen & S. C. Xiao Xiang157* (CDBI); Dêgê, Gongya township, road (highway 317) from Dêgê to Baiyu above E side of bridge over Jinsha Jiang (upper Chang Jiang) connecting Sichuan and Xizang (Tibet), downstream from confluence of Serqu He (Serqu River), dry slope above Jinsha Jiang with spinescent shrubs (ca. 1 m tall) of *Sophora*, *Rosa*, and *Caragana*, 3075 m, 31°37'40"N, 98°35'28"E, 14 August 2006, *D. E. Boufford et al. 36429* (F, PE); Dêgê, Keluodong xiang, on the way from Keluodong to Damagou, 1 August 1951, *Y. W. Tsui 5942* (PE). Xizang: Gonjo, Waba [Zeba xiang], grassland on mountain slopes, 3800 m, 20 August 1976, *Qinghai-Xizang Vegetat. Exped. 9655* (PE); Jomda, near Jomda township, on cliffs in valley, 3195 m, 31°31'39.40"N, 98°14'31.03"E, 10 August 2010, *Kham Exped. 10-1622* (PE); Jomda, Tongpu xiang, terrace in valley, 3300 m, 25 August 1973, *HNWP Xizang Exped. 2195* (HNWP).

## Supplementary Material

XML Treatment for Ajania flavida

## ﻿Additional information

### Author ORCIDs

Xiao-rui Chi https://orcid.org/0000-0002-8013-4484

Hai-song Wu https://orcid.org/0000-0003-3023-7220

Long Wang https://orcid.org/0000-0001-6059-0020

### Data availability

All of the data that support the findings of this study are available in the main text.
